# Prevalence of potentially inappropriate medication use in older population: comparison of the Finnish Meds75+ database with eight published criteria

**DOI:** 10.1186/s12877-022-03706-z

**Published:** 2023-03-10

**Authors:** Jasmin Paulamäki, Johanna Jyrkkä, Virva Hyttinen, Esa Jämsen

**Affiliations:** 1 Faculty of Medicine and Health Technology, Clinical Medicine, Tampere University, Tampere, Finland; 2 Development and Information Services, Finnish Medicines Agency, Tampere, Finland; 3 Development and Information Services, Finnish Medicines Agency, Kuopio, Finland; 4 Department of Health and Social Management, University of Eastern Finland, Kuopio, Finland; 5 Faculty of Medicine, University of Helsinki, Helsinki, Finland; 6 Department of Geriatrics, Helsinki University Hospital, Helsinki, Finland

**Keywords:** Potentially inappropriate medication list, Aged, Aged, 80 and over, Prevalence, Pharmacoepidemiology

## Abstract

**Background:**

There are several national and international criteria available for identifying potentially inappropriate medications (PIMs) for older people. The prevalence of PIM use may vary depending on the criteria used. The aim is to examine the prevalence of potentially inappropriate medication use in Finland according to the Meds75+ database, developed to support clinical decision-making in Finland, and to compare it with eight other PIM criteria.

**Methods:**

This nationwide register study consisted of Finnish people aged 75 years or older (*n* = 497,663) who during 2017–2019 purchased at least one prescribed medicine considered as a PIM, based on any of the included criteria. The data on purchased prescription medicines was collected from the Prescription Centre of Finland.

**Results:**

The annual prevalence of 10.7–57.0% was observed for PIM use depending on which criteria was used. The highest prevalence was detected with the Beers and lowest with the Laroche criteria. According to the Meds75+ database, annually every third person had used PIMs. Regardless of the applied criteria, the prevalence of PIM use decreased during the follow-up. The differences in the prevalence of medicine classes of PIMs explain the variance of the overall prevalence between the criteria, but they identify the most commonly used PIMs quite similarly.

**Conclusion:**

PIM use is common among older people in Finland according to the national Meds75+ database, but the prevalence is dependent on the applied criteria. The results indicate that different PIM criteria emphasize different medicine classes, and clinicians should consider this issue when applying PIM criteria in their daily practice.

**Supplementary Information:**

The online version contains supplementary material available at 10.1186/s12877-022-03706-z.

## Background

Inappropriate prescribing refers to the prescribing of a medication that significantly increases the risk of adverse drug events (ADEs) [1]. Potentially inappropriate medications (PIMs) are defined as medicines or medicine classes in which the risks of ADEs usually outweigh the clinical benefits and therefore these medicines should be avoided in most circumstances or for certain diseases or conditions among older people [2]. Use of PIMs has been associated with more than twofold increased odds of ADEs in people aged 65 years or older [3]. Furthermore, PIM use is associated with an increased risk of clinically important drug-drug interactions [4], hospitalizations [5] and consequently higher health care costs [6]. Despite the known risks, PIM use is common among the older population with a prevalence of 23% in Europe [7].

Several sets of criteria have been published to identify PIMs. For example, the first published criteria, the Beers criteria [2] from the United States, and two European criteria: the Laroche criteria [8] and the Screening Tool of Older Persons’ Potentially Inappropriate Prescriptions and Screening Tool to Alert to Right Treatment (STOPP/START) [9]. Later, PIM criteria have been nationally modified and published in many countries, including the Nordic countries [10–13]. However, criteria are not always applicable to other countries since PIM criteria development is country-specific with regard to prescribing practices and the available pharmaceutical market, leading to different PIM ratings. This raises concerns about their generalizability, reliability and feasibility in other settings.

Previously, several studies have compared different PIM criteria and analyzed prevalence of PIM use in various settings. For example, a prevalence range of 25–72% in PIM use has been reported when comparing the Beers criteria with STOPP criteria in studies conducted in nursing homes, hospitals and among community-dwelling older people aged 65 years or older [14–16]. Moreover, a previous nationwide register study [17] in the Swedish population aged 65 years or older compared PIMs according to five criteria and found a prevalence range of 16–-24%. Hence, it can be concluded that prevalence of PIMs varies depending on the applied criteria.

The Finnish Meds75+ database is a national database for PIM use developed to support clinical decision-making on the pharmacotherapy of people aged 75 years or over [13]. An earlier nationwide study [18] including all Finnish citizens aged 74 years or older found that the prevalence of reimbursed PIMs according to the Meds75+ database varied from 27 to 39% between Finnish hospital districts in 2011. However, the Meds75+ database has not been previously compared with other criteria.

Since the Meds75+ database and several criteria have been developed to be used in Europe, a comparison of the contents and the prevalence of PIMs is warranted. We hypothesized that different PIM criteria provide varying information about prevalence of PIM use. Therefore, our aim was to perform a cross-sectional study using nationwide register-based data to describe the prevalence of PIM use in Finland. The second aim was to provide comparing knowledge about the content of the Meds75+ database and eight other PIM criteria and to evaluate which differences in PIM rating explain the variation in prevalence.

## Methods

### Description of the Meds75+ database

The Meds75+ database is the national Finnish criteria used to identify PIMs and is maintained by the Finnish Medicines Agency Fimea [13]. The aim is to improve medication safety in primary health care and not only to classify medicines as inappropriate for persons aged 75 years or older but also to indicate medicines that are suitable or can be used with caution and is primarily intended for use by general practitioners. The database includes all drug substances or combinations used in primary health care and have at least 500 users aged 75 years or older annually. In addition, over-the-counter (OTC) medicines which are considered relevant for older persons are included. However, the database does not include substances used only in hospitals or in highly specialized care. Other PIM criteria, such as the Beers criteria, STOPP/START criteria, Laroche criteria and EU(7)-PIM list, have been used in the Meds75+ database development process [13], but the recommendations have been adjusted to the national treatment practices of Finland. The database places each drug substance in one of the following four categories: suitable for older persons (category A), present evidence or experience on use in older persons is vague or efficacy of the medicine is insufficient (B), suitable for older persons, with specific cautions (C), and avoid use in older persons (D). In addition, the database contains summarized information on the effects, dosing and most typical ADEs of the medicine. In this study, PIMs are defined according to category D. Since the database is continuously updated to consider the current prescribing practices and medicines on the Finnish market, the list of PIMs was formed on 2 July 2020.

### Other PIM criteria included

We applied the following inclusion criteria when selecting other PIM criteria for this study: (1) intended for use in older people aged 65 years or older, (2) includes drug-specific statements about medicines that should be avoided by older people in most circumstances (explicit criteria), (3) developed in Nordic countries, Europe or Northern America, and (4) published in 2006 or later (latest version included). In addition, we applied the following exclusion criteria: (1) PIM statements that are related just to certain diseases or conditions and (2) the latest publication of criteria published in 2005 or before.

First we included the four PIM criteria applied in the Meds75+ database development process (the Beers criteria, STOPP/START criteria, Laroche criteria and EU(7)-PIM list). Furthermore, in order to focus on the Meds75+ database and prescribing guidelines in Northern Europe, we searched the literature and utilized reviews [19–21] to select criteria from other Nordic countries. Moreover, to be able to evaluate the classification discrepancies across Europe, we included the PRISCUS (Latin for “old and venerable”) list [22] since the preliminary EU(7)-PIM list also contained PIMs from the German criteria [23]. Altogether, a total of nine sets of PIM criteria were included and are described in Table 1.

**Table 1:** Description of the nine PIM criteria used in this study

Criteria (country of origin)	Number of PIM statements	Age group	Year of the first published version	Year of the latest update
Meds75+ database [13] (Finland)	91	≥75	2010	Continuously updated
Beers criteria [24] (USA)	46	≥65	1991	2019
EU(7)-PIM list [23] (Estonia, Finland, France, Germany, the Netherlands, Spain and Sweden)	282	≥65^a^	2015	2015
Indicators for Quality of Drug Therapy in the elderly [12] (Sweden)	9	≥75	2004	2017
Laroche criteria [8] (France)	34	≥75	2007	2007
NORGEP-NH [25] (Norway)	34	≥70	2009	2015
PRISCUS list [22] (Germany)	83	≥65	2010	2010
Red-Yellow-Green list [26] (Denmark)	44	≥65	2011	2016
STOPP(/START) [27] (Ireland)	114	≥65	2008	2015

*NH* nursing home, *NORGEP* Norwegian General Practice, *PIM* potentially inappropriate medication, *STOPP/START* Screening Tool of Older Persons’ Potentially Inappropriate Prescriptions and Screening Tool to Alert to Right Treatment

^a^preliminary PIM list was developed, based on other PIM lists; all targeted persons aged 65 years or older

The latest version of the Beers criteria [24] is divided into five sections, of which *medications that are potentially inappropriate in most older adults* were considered as PIMs in this study. Similarly, the following statement, from the nine sets of Swedish quality indicators for evaluation of older patients’ drug therapies (later the Swedish criteria) [12] were considered as PIMs: *medicines that should be avoided unless there is a special reason for using them*. The STOPP/START criteria [27] also include potential prescribing omissions in the START criteria, so in this study we only considered a list of PIMs in the STOPP criteria. Although, since STOPP criteria includes many statements considering PIM use in specific medical conditions, requiring clinical information, we applied the statements partially.

From the Norwegian General Practice (NORGEP) criteria [10], we used part A (regular use should be avoided) of the version updated in 2015 for assessing medication for nursing home residents (NORGEP-NH) [25], since it is based on the original NORGEP criteria and a comprehensive literature search. From the Danish Red-Yellow-Green list, drugs in the red category (drugs that should not be used in older people) were considered as PIMs. Although the Danish list was updated in 2016, the original edition was selected, since it has been published in English with ATC codes [26] and therefore is more accurate and leaves no room for interpretation.

### Identifying potentially inappropriate medications

Drug-specific PIM statements were extracted from each of the nine criteria and entered into an Excel file. One author (JP) examined the selected criteria and created the summary table. If the criteria included a statement to avoid a medicine class, the statement was documented in the summary for medicines mentioned by name in other criteria. For example, the NORGEP-NH includes a statement about regular use of hypnotics. According to the Anatomical Therapeutic Chemical (ATC) classification system by the World Health Organization [28], the code for hypnotics and sedatives is N05C. Based on this international drug classification, the statement from NORGEP-NH was documented for drugs whose ATC code is N05C and those listed in other included criteria. Unclear classifications were discussed among the three authors (JP, JJ, EJ).

We did not consider the following kinds of PIM statements from the final table: (1) concurrent use of two or more drugs (e.g., warfarin combined with non-steroidal anti-inflammatory drugs (NSAIDs)), (2) PIMs and specific conditions (e.g., Angiotensin Receptor Blockers in patients with hyperpotassemia), (3) PIMs and restriction of treatment duration or dose, (4) PIMs with limited research evidence or experience among older people, and (5) anticholinergic medicines without a specific active substance. Not including a statement was related to the restrictions on the data. First, the statements requiring additional clinical information (e.g., diagnosis, renal insufficiency, dosage, treatment duration) were not applied since such details could not be captured from the national prescription register. Second, some of the included criteria (e.g., Meds75+ database, Beers criteria) contain a special category for medicines with specific caution (e.g. use with caution, present evidence or experience with use in older persons is vague). Overall, the comparison of criteria focused on identifying PIMs to avoid generally without considering additional clinical information. This research strategy collected a summary of 352 ATC codes considered as PIMs (see Additional file 1).

The summary was screened using both the Social Insurance Institution (SII) of Finland’s Medicinal Products Database and Finnish Medicines Agency’s FimeaWeb to exclude medicines not available in Finland. If the medicine was available on the market only in combination (e.g., codeine), the medicine was still included. In addition, if the medicine was available both as a single active substance and in combination (e.g., oxycodone), both ATC codes were included. Finally, after screening the summary, 172 ATC codes were considered as PIMs available on Finnish pharmaceutical market (see Additional file 1).

### Data collection and statistical analysis

Since the Meds75+ database is intended to be used for older people aged 75 years or older this age limit was applied in the study population. In this retrospective cross-sectional study, the study population was drawn from the entire population of older people aged 75 years or older in Finland in 2017–2019. We obtained data on purchased prescription medicines, which reflects PIM use better than prescriptions only. The Prescription Centre of Finland is a national register held by the SII and contains all human medicine prescriptions and their medicine purchases delivered from pharmacies in Finland since 2017 [29, 30]. The database covers all Finnish citizens except persons living permanently in institutions (< 1% of persons aged ≥75 years) and medicines given in hospitals. The study cohort consisted of older people who had purchased at least one prescription medicine, reimbursed by National Health Insurance (tax-supported public social security coverage) or not, considered as a PIM (see Additional file 1) in 2017–2019 and were aged 75 years or over at the time of purchase. In order to calculate the total number of PIMs purchased by an individual, we used a pseudonymized identification number for every person.

Altogether the data consisted of 523,263 older person and their 14,488,277 medicine purchases from 1 January 2017 to 31 December 2019. To allow comparison between study years, the data was split into three cohorts, one for each year. After exclusion of prescription purchases with incorrect age criteria, missing gender, ATC code not included in the PIM summary table or invalid pharmaceutical form (e.g. topical estrogen), the final data used to assess the prevalence of PIMs in Finland consisted of 497,663 older people and their 11,685,648 medicine purchases.

The population characteristics were presented as mean values with standard deviations. The prevalence of PIM use was calculated based on census data obtained from Statistics Finland (mean population aged 75 years or older in 2017: *N* = 500,820.5; 2018: *N* = 506,884.5; and 2019: *N* = 518,276.0) [31]. The annual prevalence was calculated by dividing the number of older persons with at least one PIM purchase by the average population of each year. The prevalence of PIM use was presented as annual percentages per criteria. The number of PIMs per person was calculated as each individual ATC code regardless of the number of purchases. We also reported the most commonly used PIM classes and their prevalence per criteria during the three-year observation period. In addition, the most commonly used PIMs per criteria were presented. For numerical values, relevant descriptive statistics were presented (mean, standard deviation, median). The statistical analyses were performed using the IBM© SPSS© Statistics software, version 26.

## Results

A total of 497,663 older people who had purchased altogether 168 different PIMs were included. The mean age of the population was 82.6 ± 5.7 years, and two thirds (61.8%) were female.

### Prevalence of PIM use

We observed an annual prevalence of PIM use of 10.7–57.0% for Finnish older persons according to different criteria (Table 2). According to the Meds75+ database, the annual prevalence of PIM use was 30.4–33.6%. The Beers criteria and STOPP criteria resulted in higher prevalences (annually over 50%) than the other criteria (the third highest prevalence, with EU(7)PIM list, was 39.9% in 2017). Regardless of the applied criteria, the annual prevalence of PIM use decreased during the three-year period, from 14.2–57.0% in 2017 to 10.7–55.3% in 2019.

**Table 2:** Number of identified PIMs and annual prevalence of use according to different criteria

Criteria	PIMs^**a**^ identified	PIMs per person	Prevalence in 2017, %	Prevalence in 2018, %	Prevalence in 2019, %	Overlap between Meds75+ and other criteria %^**b**^
Meds75+ database (Finland)	91	1–13	33.6	32.7	30.4	–
Beers criteria (USA)	79	1–17	57.0	56.4	55.3	57.4
EU(7)-PIM list (Estonia, Finland, France, Germany, the Netherlands, Spain and Sweden)	128	1–16	39.9	38.9	37.5	72.2
Indicators for Quality of Drug Therapy in the elderly (Sweden)	28	1–8	17.0	16.2	15.7	99.1
Laroche criteria (France)	38	1–7	14.2	13.1	10.7	95.2
NORGEP-NH (Norway)	29	1–11	36.0	34.9	33.7	65.3
PRISCUS list (Germany)	35	1–8	24.0	22.9	20.6	72.2
Red-Yellow-Green list (Denmark)	38	1–9	30.1	29.6	28.9	56.9
STOPP(/START) (Ireland)	65	1–17	52.1	51.7	50.8	57.9

*NH* nursing home, *NORGEP* Norwegian General Practice, *PIM* potentially inappropriate medication, *STOPP/START* Screening Tool of Older Persons’ Potentially Inappropriate Prescriptions and Screening Tool to Alert to Right Treatment

^a^available on Finnish pharmaceutical market

^b^proportion of persons identified as PIM users consistently by the Meds75+ database and the other criterion, compared to the total number of PIM users in 2017–2019 of the other criterion

The number of PIMs per person varied between 1 and 17 according to the different criteria (Table 2). Depending on the applied criteria, proportion of 3.8–45.5% of older people with at least one PIM purchase used two or more PIMs during the observation period. The overlap between the Meds75+ database and eight criteria in detecting PIM users varied from 56.9 to 99.1%. The overlap was greatest between the Meds75+ database and the Swedish criteria, whereas only less than two third of PIM users according to the Beers criteria, STOPP criteria and the Red-Yellow-Green list were also identified by Meds75+ database.

### Variation in PIM ratings

The most common medicine classes considered as PIMs were benzodiazepines (N05BA and N05CD), tricyclic antidepressants (N06AA) and drugs for urinary frequency and incontinence (G04BD). Considering individual drug substances, only amitriptyline, clomipramine and diazepam were considered as PIMs in all nine criteria. Most of the criteria identified also hydroxyzine (8/9), levomepromazine (8/9), nitrazepam (8/9), indomethacin (7/9), nortriptyline (7/9) solifenacin (7/9), tolterodine (7/9), and trimipramine (7/9).

Differences in the proportions of PIM users was observed in medicine classes (Table 3) and individual drug substances (see Additional file 2) when used different PIM criteria. For example, when the Beers criteria is applied, proton pump inhibitors (PPIs) were the most commonly used PIM class (46.4% of the total study population *n* = 497,663 with at least one PIM). When the STOPP criteria was applied the most commonly used PIM classes were loop diuretics (33.5%) and opioids (28.4%). These medicines were considered as PIMs only in one criterion, which led to higher prevalence when that criterion was applied. In case PPIs are not considered, the annual prevalences of PIM use according to the Beers criteria were as follows: 44.1% (2017), 42.7% (2018) and 40.8% (2019). According to STOPP criteria these prevalences were about 10 percentage points lower if loop diuretics and all opioids except codeine were not considered: 36.6% (2017), 35.4% (2018) and 34.0% (2019). Moreover, if these medicines are not considered, the most commonly used PIM class was found to be NSAIDs (30.1%) according to the NORGEP-NH and Red-Yellow-Green list.

**Table 3:** Proportion of users of the most common PIMs among people using PIMs (total = 497,663)

ATC code and medicine subgroup	All criteria considered^**a**^ (%)	Number of criteria defining PIM class^**b**^	Meds75+ database (%)	Beers criteria (%)	EU (7)-PIM list (%)	Swedish criteria^**c**^ (%)	Laroche criteria (%)	NORGEP-NH (%)	PRISCUS list (%)	Red-Yellow-Green list (%)	STOPP (/START) (%)
A02B Drugs for peptic ulcer and gastro-oesophageal reflux disease	46.8	2/9	0.8	**46.4**							
N02A Opioids	38.1	5/9	**20.5**		6.6	**20.5**		16.0			28.4
C03C High-ceiling diuretics/Loop diuretics	33.5	1/9									**33.5**
M01A Anti-inflammatory products, non-steroids	30.1	7/9	0.4	22.8	**16.0**		0.4	**30.1**	**12.1**	**30.1**	
N05C Hypnotics and sedatives	23.4	9/9	3.8	9.7	7.0	0.3	0.3	23.4	6.9	0.3	23.4
N05B Anxiolytics	17.6	9/9	6.2	17.5	6.3	5.4	**5.5**	5.4	6.3	3.0	17.6
N05A Antipsychotics	14.4	8/9	1.9	14.4	1.4	0.8	0.6		0.7	9.0	14.4

**bold number** the medicine class with highest proportion of users per each criterion

*NH* nursing home, *NORGEP* Norwegian General Practice, *PIM* potentially inappropriate medication, *STOPP/START* Screening Tool of Older Persons’ Potentially Inappropriate Prescriptions and Screening Tool to Alert to Right Treatment

^a^potentially inappropriate medications according to the summary of nine criteria

^b^even if the same medicine class is included in several PIM criteria, the drug substances considered as PIMs may differ between the criteria, leading to variation in the reported percentages (see Additional file 2)

^c^Indicators for Quality of Drug Therapy in the elderly

Of individual drug substances, the most commonly used PIMs were furosemide, pantoprazole, and codeine. Included nine criteria rated the medicines quite similarly with regard to the most commonly used PIMs (Table 4). Opioids and NSAIDs were among the top three PIMs in most criteria.

**Table 4:** The top three PIMs and proportion of users among people using PIMs (total = 497,663)

Criteria	Drug substance	Proportion of users (%)^**a**^
Meds75+ database (Finland)	Codeine	16.0
	Tramadol	6.6
	Nitrofurantoin	6.5
Beers criteria (USA)	Pantoprazole	33.0
	Ibuprofen	15.3
	Oxazepam	10.2
EU(7)-PIM list (Estonia, Finland, France, Germany, the Netherlands, Spain and Sweden)	Etoricoxib	9.7
	Temazepam	6.6
	Tramadol	6.6
Indicators for Quality of Drug Therapy in the elderly (Sweden)	Codeine	16.0
	Tramadol	6.6
	Amitriptyline	4.0
Laroche criteria (France)	Nitrofurantoin	6.5
	Amitriptyline	4.0
	Diazepam	3.0
NORGEP-NH (Norway)	Combination of Codeine and paracetamol	16.0
	Ibuprofen	15.3
	Zopiclone	14.7
PRISCUS list (Germany)	Etoricoxib	9.7
	Temazepam	6.6
	Nitrofurantoin	6.5
Red-Yellow-Green list (Denmark)	Ibuprofen	15.3
	Etoricoxib	9.7
	Quetiapine	7.0
STOPP(/START) (Ireland)	Furosemide	33.5
	Oxycodone	15.6
	Zopiclone	14.7

*NH* nursing home, *NORGEP* Norwegian General Practice, *PIM* potentially inappropriate medication, *STOPP/START* Screening Tool of Older Persons’ Potentially Inappropriate Prescriptions and Screening Tool to Alert to Right Treatment

^a^among persons with at least one PIM in use during the observation period

## Discussion

This is the first study describing and comparing nationwide prevalence of PIM use according to the Finnish Meds75+ database and eight different sets of PIM criteria. Our results indicate that the prevalence varied significantly (10.7–57.0%) between the different criteria among Finnish people aged 75 years or older during the three-year period. The highest prevalence of PIM use was detected with the Beers criteria and the lowest with the Laroche criteria. The annual prevalence detected with the Meds75+ database, one-third, was in the middle of the values obtained by other criteria. Also, the number of medications considered as PIMs and the overlap between the Meds75+ database and eight criteria varied clearly. Depending on the applied criteria, the proportion of people who used more than one PIM over the whole study period varied from 3.8 to 45.5%. However, the annual prevalence of PIMs decreased during the years 2017–2019 regardless of the applied criteria.

We hypothesized that different criteria used to evaluate PIMs provide varying information about their prevalence, and this is supported by our findings. The observation that the Beers and STOPP criteria detect higher PIM prevalence than other included European PIM criteria is consistent with earlier studies comparing more than four criteria [17, 32]. However, findings considering the variance of prevalence are mixed. A study comparing six criteria found a similar high variance, from 24% (NORGEP) to 63% (STOPP), among people aged 65 years or older in Taiwan [32]. On the other hand, a Swedish study detected PIM use by applying five criteria and found a smaller variance from 16.0% (NORGEP) to 24.1% (Beers) [17]. The mixed results might also demonstrate the fact that the criteria are not equally applicable since they are originally intended for use among different age groups. Furthermore, some of the criteria are older than others which may effect on sensitivity in detecting PIMs. These results support the finding from previous study [33] comparing EU(7)PIM and PRISCUS list, which concluded that differences between listed PIMs support regular updating of the PIM criteria.

According to the Meds75+ database, annually one third of Finnish older persons had used PIMs. A similar prevalence was also found in a study applying the Meds75+ database in a smaller population (*n* = 1000) with the same age limit [34], although that study determined the medications by interviews and included both prescription and OTC medicines. It should also be noted that the Meds75+ database is continuously updated, and the list of PIMs and prescribing practices have changed over time.

We found that annually over half of older persons used PIMs according to the Beers criteria. This prevalence is significantly higher compared to an earlier Finnish population-based register study which found a prevalence of 15% according to the Beers criteria [35]. The study population in that study also included Finnish people aged 65 years or older and the data consisted of only prescription PIMs reimbursed by National Health Insurance, which may explain the difference in prevalence compared to our results.

Our prevalences with the Meds75+ database and NORGEP-NH are consistent with the Norwegian cross-sectional study among home-dwelling people aged 70 years or older, according to the national NORGEP criteria [36]. On the other hand, we report higher prevalence with our national PIM criteria compared to the studies in the Swedish population with their national criteria (17–19%) [17, 37]. However, their prevalence is consistent with ours according to the Swedish criteria. In contrast with Norway and Sweden, we found a lower prevalence of PIM use with both the Meds75+ database and the Red-Yellow-Green list than a study conducted in the Danish population aged 65 years or older [38]. However, the Danish study also found that prevalence of PIM use decreased during the follow-up, which is consistent with our result.

The finding that annual PIM prevalence is decreasing is in concordance with two earlier Finnish studies. First, a register-based study reported that use of reimbursed PIMs decreased from 43 to 18% between 2000 and 2013 [39]. More recently, The Finnish Medicines Agency Fimea, who follows and compiles nationwide statistics about the quality of care of people aged 75 or older, reported that the decline had continued in 2017–2019, but still one fifth of older persons used PIMs, based on the Med75+ database [40]. The lower prevalence in these reports is most likely due to the restriction of PIMs to only prescription purchases reimbursed by National Health Insurance, whereas our data included also non-reimbursed prescription purchases. Moreover, the earlier study included also people aged 65–74 that may explain the higher prevalence at the beginning of the follow-up time compared to ours.

Based on our study, the differences in PIM prevalence are explained by the variances in prevalence of certain medicine classes. We found that more than one fifth of older persons had purchased a PPI, opioid, loop diuretic, NSAID or hypnotic, but the proportion of users of these medicine classes is dependent on the applied criteria. From this perspective, our results suggest that different criteria emphasize different medicine classes in their definition of PIMs. The high prevalences of PIM use detected with the Beers criteria and STOPP criteria is partially explained by the common use of PPIs, furosemide and opioids. If these medicines are not considered, the detected prevalence decreases almost to the same level as the Meds75+ database. Moreover, the Finnish Meds75+ database classifies PPIs and furosemide as suitable for older people and therefore these medicines are considered as a safe therapy option in Finland. Furthermore, the Beers criteria and STOPP criteria consider anxiolytics and antidepressants more comprehensively as PIMs compared to the other seven criteria, which also increases the prevalence according to these two PIM criteria.

Three medicines, amitriptyline, clomipramine and diazepam, were considered as PIMs in all criteria, which is consistent with findings from an earlier study [17]. Furthermore, we found that the nine criteria identify the most-used PIMs quite similarly. This is in contrast with results of an earlier study [32] which found that the top three PIMs varied significantly between the six criteria. However, they analyzed different criteria and had a small sample size (*n* = 193) which affects comparability. We detected codeine (Meds75+ database, Swedish criteria and NORGEP-NH), etoricoxib (EU(7)PIM list, PRISCUS list and Red-Yellow-Green list), ibuprofen (Beers criteria, NORGEP-NH and Red-Yellow-Green list), nitrofurantoin (Meds75+ database, Laroche criteria and PRISCUS list) and/or tramadol (Meds75+ database, EU(7)PIM list and Swedish criteria) as the most-used PIMs in three different criteria. This might be explained by the relation between the nine criteria, since the authors and experts developing and updating national criteria generally use PIM criteria from other countries.

### Strengths and limitations

The present study has several strengths. Unlike earlier studies comparing PIM criteria, this study compiled all PIMs at population level regardless of, for example, comorbidities, and used nine different criteria, including the Finnish Meds75+ database. In addition, this is the first study comparing the content of the Meds75+ database to eight criteria in identifying PIMs. Furthermore, the very high coverage of the national prescription register makes it possible to obtain generalizable findings.

On the other hand, certain limitations should be noted. First, seven medicines were misinterpreted (i.e. interpreted as PIM only in certain clinical conditions or questionable PIM) from the EU(7)-PIM list and were not included in the data collection (see Additional file 1). Consequently, the actual prevalence of PIM use according the EU(7)-PIM list was underestimated. However, the effect of misinterpretation on the prevalence can be expected to be small based on the Finnish Statistics on Medicines published by the Finnish Medicines Agency Fimea and the SII [41].

Second, since different criteria rate PIMs at different levels, our comparison of nine criteria is limited. For example, medicines with anticholinergic burden have not been internationally defined but the Laroche criteria includes a statement to avoid concomitant use of anticholinergic drugs without specific drug substance. Although the Beers criteria, the Meds75+ database and the Swedish criteria consider individual drug substances with anticholinergic properties as PIMs, the Laroche statement was not considered for these drugs listed in other included criteria. The lower prevalence of PIM use detected with the Laroche criteria may be explained with the excluded anticholinergics.

Third, information on purchases rather than actual intake of medicines is a limitation. However, analysis of actual intake at nationwide scale is hardly possible. Furthermore, we could not take OTC medicines into account as the Prescription Centre does not register their use. As some PIMs are available also without prescription in Finland (e.g., acetylsalicylic acid), their actual use may be greater than we have observed. On long-term use, having a prescription makes the use cheaper so the significance of missing data about short-term OTCs is probably relatively limited.

Forth, we did not take into account those cases where PIM status of a medicine is limited to certain doses, indications, types of pharmaceutical form or duration of treatment. Although the Prescription Centre includes detailed information about deliveries (incl. For example the package size), dosage instructions are not recorded in such systematic way that would allow consideration of dosages or indications and collecting such information manually from the extensive data would have been unnecessarily challenging. For these reasons, the prevalence of PIM use may be undersized with the criteria where PIM status depends on these type of details related to the treatment: NORGEP-NH, STOPP, Red-Yellow-Green list, EU(7)PIM list and Laroche criteria.

Fifth, although the included criteria are intended for use in older persons, the age limit for this age group varies. Different age limits may affect which medications are considered as PIMs and thereby partly explain the variation in the observed prevalences. As the medicines that are considered as PIMs for older persons aged 65 or over should - quite obviously – be avoided in persons aged 75 years or over as well, all included criteria can be applied to our study population.

## Conclusion

Our findings indicate that PIM use is common among older people in Finland according to the national Meds75+ database, but the prevalence varies significantly between the applied nine criteria. Furthermore, this study underlines the focus of the different criteria that clinicians should consider when applying PIM criteria in daily practice. Although we found that the nine criteria detect the most-used individual PIMs quite similarly, the differences in the prevalence of medicine classes explain the variation in overall prevalence. Differences in defining PIMs between criteria must be considered when studying use of medications in different areas and health care settings. Future research should focus on evaluating how medicine use is affected by the different criteria over time.

## Supplementary Information

**Additional file 1.** Complete summary of PIMs, ignored statements and PIMs not available on the Finnish market.

**Additional file 2.** Proportion of users of the most common medicine classes/substances among people using PIMs (total = 497,663).

## Data Availability

The data that support the findings of this study are available from The Social Insurance Institution of Finland but restrictions apply to the availability of these data, which were used under license for this study, and so are not publicly available. Data are available from The Social Insurance Institution of Finland with the permission of The Finnish Social and Health Data Permit Authority Findata.

## Abbreviations

ADE: Adverse drug event
PIM: Potentially inappropriate medication
STOPP/START: Screening Tool of Older Persons’ Potentially Inappropriate Prescriptions and Screening Tool to Alert to Right Treatment
OTC: Over-the-counter
PRISCUS: Latin for “old and venerable”
Swedish criteria: Swedish quality indicators for evaluation of older patients’ drug therapies
NORGEP: Norwegian General Practice
NH: Nursing home
ATC: Anatomical Therapeutic Chemical
NSAIDs: Non-steroidal anti-inflammatory drugs
SII: Social Insurance Institution of Finland
PPIs: Proton pump inhibitors
N05BA and N05CD: World Health Organization’s Anatomical Therapeutic Chemical classification system for benzodiazepines
N06AA: World Health Organization’s Anatomical Therapeutic Chemical classification system for tricyclic antidepressants
G04BD: World Health Organization’s Anatomical Therapeutic Chemical classification system for drugs for urinary frequency and incontinence
