# Supplementary material for: Prevalence of potentially inappropriate medication use in older population: comparison of the Finnish Meds75+ database with eight published criteria
Source: BMC Geriatr. 2023 Mar 10;23:139. doi: 10.1186/s12877-022-03706-z (PMC9999502; doi:10.1186/s12877-022-03706-z)
Supplement: Supplementary file 1 — Additional file 1. Complete summary of PIMs, ignored statements and PIMs not available on the Finnish market. [file 12877_2022_3706_MOESM1_ESM.docx]

Prevalence of potentially inappropriate medication use in older population: comparison of the Finnish Meds75+ database with eight published criteria

**Journal name:** BMC Geriatrics

**Author names:** Paulamäki Jasmin, Jyrkkä Johanna, Hyttinen Virva, Jämsen Esa

**Corresponding author:** Jasmin Paulamäki, Faculty of Medicine and Health Technology, Clinical Medicine, Tampere University, Tampere, Finland. Email: jasmin.paulamaki@tuni.fi

**Description of the data**: Table of the complete summary of PIMs (P) in nine included criteria, ignored statements (I) and potentially inappropriate medications not available on the Finnish market (M).

**Abbreviations**: ^1^: misinterpreted from the EU(7)-PIM list and not included in the data collection; I: excluded statement in order to form a table of medications that should be avoided by older people in most circumstances: statements considering concurrent use of two or more drugs, specific medical condition, restriction of treatment duration or dose, limited research evidence or experience among older people; M: potentially inappropriate medication not available on Finnish market; NH: nursing home; NORGEP: Norwegian General Practice; P: considered as potentially inappropriate medication; PIM: potentially inappropriate medication; STOPP/START: Screening Tool of Older Persons’ Potentially Inappropriate Prescriptions and Screening Tool to Alert to Right Treatment

**Additional file 1.** Complete summary of PIMs, ignored statements and PIMs not available on the Finnish market.

| **ATC (according**  **to WHO [28]**  **ATC code)** | **Active substance** | **Ignored**  **statement;**  **excluded**  **from**  **the data**  **collection** | **Not available on Finnish market** | **Meds75+ 2020 [13]** | **Indicators for Quality of Drug Therapy in the elderly 2017 [12]** | **NORGEP-NH 2015 [25]** | **Beers criteria 2019 [24]** | **STOPP/**  **(START) 2015 [27]** | **Red-Yellow-Green list 2011 [26]** | **EU(7)-PIM 2015 [23]** | **PRISCUS list 2010 [22]** | **Laroche criteria 2007 [8]** |
| --- | --- | --- | --- | --- | --- | --- | --- | --- | --- | --- | --- | --- |
| A02AA04 | Magnesium hydroxide |  |  | P |  |  |  |  |  | P |  |  |
| A02AB | Aluminium compounds | x | x |  |  |  |  | I |  | M |  |  |
| A02AD | Aluminium Combinations and complexes of aluminium, calcium and magnesium compounds |  |  | P |  |  |  | I |  | I |  |  |
| A02BA01 | Cimetidine | x | x |  |  |  |  |  |  | M |  | M |
| A02BA02 | Ranitidine | x | x |  |  |  |  |  |  | M |  |  |
| A02BA03 | Famotidine |  |  |  |  |  |  |  |  | P |  |  |
| A02BA53 | Famotidine, combinations |  |  | P |  |  |  |  |  |  |  |  |
| A02BC01 | Omeprazole |  |  |  |  |  | P | I |  | I |  |  |
| A02BC02 | Pantoprazole |  |  |  |  |  | P | I |  | I |  |  |
| A02BC03 | Lansoprazole |  |  |  |  |  | P | I |  | I |  |  |
| A02BC04 | Rabeprazole |  |  |  |  |  | P | I |  | I |  |  |
| A02BC05 | Esomeprazole |  |  |  |  |  | P | I |  | I |  |  |
| A02BX02 | Sucralfate |  |  | P |  |  |  |  |  |  |  |  |
| A02BX13 | Alginic acid |  |  | P |  |  |  |  |  |  |  |  |
| A03AA04 | Mebeverine | x | x |  |  |  |  |  |  | M |  |  |
| A03AA05 | Trimebutine | x | x |  |  |  |  |  |  | M |  |  |
| A03AA07 | Dicycloverine | x | x |  |  |  | M |  |  |  |  |  |
| A03AA08 | Dihexyverine | x | x |  |  |  |  |  |  | M |  | M |
| A03AB02 | Glycopyrronium bromide |  |  |  | P |  |  |  |  |  |  |  |
| A03AB05 | Propantheline | x | x |  |  |  | M |  | M |  |  |  |
| A03AB06 | Otilonium bromide | x | x |  |  |  |  |  |  | M |  |  |
| A03AB17 | Tiemonium iodide | x | x |  |  |  |  |  |  | M |  | M |
| A03AX04 | Pinaverium | x | x |  |  |  |  |  |  | M |  |  |
| A03BA01 | Atropine | x | x |  | M |  | M |  |  | M |  | M |
| A03BA03 | Hyoscyamine | x | x |  | M |  | M |  |  | M |  |  |
| A03BA04 | Belladonna total alkaloids | x | x |  |  |  | M |  |  | M |  | M |
| A03BB01 | Butylscopolamine |  |  |  | P |  |  |  | P |  |  |  |
| A03BB03 | Methylscopolamine | x | x |  | M |  | M |  |  |  |  |  |
| A03CA02 | Clidinium and psycholeptics |  |  | P |  |  | P |  |  | P |  | P |
| A03DA02 | Pitofenone |  |  | P |  |  |  |  |  | P |  |  |
| A03FA01 | Metoclopramide |  |  | P |  |  |  | I | I | P |  |  |
| A03FA03 | Domperidone | x | x |  |  |  |  |  |  | I |  |  |
| A03FA05 | Alizapride | x | x |  |  |  |  |  |  | M |  | M |
| A04AB02 | Dimenhydrinate | x | x |  |  |  | M | M |  | M | M | M |
| A04AD01 | Scopolamine |  |  | P | P |  | P |  |  | P |  | P |
| A04AD05 | Metopimazine | x | x |  |  |  |  |  |  | M |  | M |
| A06AA01 | Liquid paraffin | x | x |  |  |  | M |  |  | M | M |  |
| A06AA02 | Docusate sodium | x | x |  |  |  |  |  |  | M |  | M |
| A06AB02 | Bisacodyl |  |  | P |  |  |  |  | P | I |  | P |
| A06AB05 | Castor oil | x | x |  |  |  |  |  |  | M |  | M |
| A06AB06 | Senna glycosides |  |  | P |  |  |  |  |  | P |  | P |
| A06AB07 | Cascara sagrada | x | x |  |  |  |  |  |  | M |  | M |
| A06AB08 | Sodium picosulfate |  |  | P |  |  |  |  | P | P |  | P |
| A06AB13 | Aloe | x | x |  |  |  |  |  |  | M |  | M |
| A06AB56 | Senna glycosides, combinations |  |  | P |  |  |  |  |  | P |  | P |
| A06AB58 | Sodium picosulfate, combinations |  |  | P |  |  |  |  | P | P |  | P |
| A06AG02 | Bisacodyl |  |  | P |  |  |  |  |  |  |  | P |
| A06AG10 | Docusate sodium |  |  |  |  |  |  |  |  |  |  | P |
| A06AX05 | Prucalopride | x^1^ |  |  |  |  |  |  |  | P |  |  |
| A07DA01 | Diphenoxylate | x | x |  |  |  |  |  |  | M |  | M |
| A07DA03 | Loperamide | x |  |  |  |  |  |  |  | I |  |  |
| A07XA04 | Racecadotril | x |  |  |  |  |  |  |  | I |  |  |
| A10AB01 | Insulin (human) |  |  |  |  |  | P |  |  | P |  |  |
| A10AB03 | Insulin (pork) |  |  |  |  |  | P |  |  | P |  |  |
| A10AB04 | Insulin lispro |  |  |  |  |  | P |  |  | P |  |  |
| A10AB05 | Insulin aspart |  |  |  |  |  | P |  |  | P |  |  |
| A10AB06 | Insulin glulisine |  |  |  |  |  | P |  |  | P |  |  |
| A10BB01 | Glibenclamide | x | x |  | M |  | M | M |  | M |  |  |
| A10BB02 | Chlorpropamide | x | x |  |  |  | M | M |  | M |  |  |
| A10BB06 | Carbutamide | x | x |  |  |  |  |  |  | M |  | M |
| A10BB07 | Glipizide | x | x |  |  |  |  |  |  | M |  | M |
| A10BB12 | Glimepiride |  |  | P |  |  | P | P |  | P |  |  |
| A10BD09 | Pioglitazone and alogliptin |  |  |  |  |  |  | I |  | P |  |  |
| A10BF01 | Acarbose | x | x |  |  |  |  |  |  | M |  |  |
| A10BG03 | Pioglitazone |  |  |  |  |  |  | I |  | P |  |  |
| A10BH01 | Sitagliptin | x |  |  |  |  |  |  |  | I |  |  |
| A10BH02 | Vildagliptin | x |  |  |  |  |  |  |  | I |  |  |
| A10BK01 | Dapagliflozin |  |  | P |  |  |  |  |  |  |  |  |
| A10BX02 | Repaglinide |  |  | P |  |  |  |  |  |  |  |  |
| A12BA51 | Potassium chloride, combinations |  |  | P |  |  |  |  |  |  |  |  |
| B01AA07 | Acenocoumarol | x | x |  |  |  |  |  |  | M |  |  |
| B01AC05 | Ticlopidine | x | x |  |  |  |  | M |  | M | M | M |
| B01AC06 | Acetylsalicylic acid | x |  |  |  |  |  | I | I |  |  |  |
| B01AC07 | Dipyridamole |  |  |  |  |  | P | I |  | P |  | P |
| B01AC22 | Prasugrel |  |  |  |  |  |  |  |  | P | P |  |
| B01AC30 | Combinations |  |  |  |  |  |  |  | P |  |  |  |
| B01AE07 | Dabigatran etexilate | x |  |  |  |  |  |  |  | I |  |  |
| B01AF01 | Rivaroxaban | x |  |  |  |  |  |  |  | I |  |  |
| B01AF02 | Apixaban | x |  |  |  |  |  |  |  | I |  |  |
| B03AA | Iron preparations | x |  |  |  |  |  | I |  | I |  |  |
| C01AA02 | Acetyldigoxin | x | x |  |  |  |  |  |  | M | M |  |
| C01AA04 | Digitoxin | x | x |  |  |  |  |  |  | M |  |  |
| C01AA05 | Digoxin |  |  |  |  |  | P | I | P | P | P | I |
| C01AA08 | Metildigoxin | x | x |  |  |  |  |  |  | M | M |  |
| C01BA01 | Quinidine | x | x |  |  |  |  |  |  | M | M |  |
| C01BA02 | Procainamide | x | x |  |  |  |  |  |  | M |  |  |
| C01BA03 | Disopyramide |  |  |  | P |  | P |  |  | P |  | P |
| C01BA51 | Quinidine, combinations excl. psycholeptics | x | x |  |  |  |  |  |  | M |  |  |
| C01BC03 | Propafenone | x | x |  |  |  |  |  |  | M |  |  |
| C01BC04 | Flecainide |  |  | P |  |  |  |  |  | P | P |  |
| C01BD01 | Amiodarone |  |  | P |  |  | P | P |  | P |  |  |
| C01BD07 | Dronedarone |  |  |  |  |  | P |  |  | P |  |  |
| C01EB15 | Trimetazidine | x | x |  |  |  |  |  |  | M |  |  |
| C01EB17 | Ivabradine | x^1^ |  |  |  |  |  |  |  | P |  |  |
| C02AA02 | Reserpine | x | x |  |  |  | I |  |  | M | M | M |
| C02AB01 | Methyldopa | x | x |  |  |  | M | M |  | M | M | M |
| C02AC01 | Clonidine |  |  |  |  |  | P | P |  | P | P | P |
| C02AC02 | Guanfacine |  |  |  |  |  |  | P |  | P |  | P |
| C02AC05 | Moxonidine |  |  | P |  |  |  | P |  | P |  | P |
| C02AC06 | Rilmenidine | x | x |  |  |  |  | M |  | M |  | M |
| C02CA01 | Prazosin |  |  | P |  |  | P |  |  | P | P | I |
| C02CA04 | Doxazosin | x | x |  |  |  | M |  |  | M | M |  |
| C02CA06 | Urapidil | x | x |  |  |  |  |  |  | M |  | I |
| C02CC02 | Guanethidine | x | x |  |  |  |  |  |  | M |  |  |
| C02DB02 | Hydralazine | x | x |  |  |  |  |  |  | M |  |  |
| C03CA01 | Furosemide |  |  |  |  |  |  | P | I |  |  |  |
| C03DA01 | Spironolactone | x |  |  |  |  |  | I |  | I |  |  |
| C03EB01 | Furosemide and potassium-sparing agent |  |  |  |  |  |  | P | I |  |  |  |
| C04AA01 | Isoxsuprine | x | x |  |  |  | M |  |  |  |  |  |
| C04AD03 | Pentoxifylline |  |  | P |  |  |  |  |  | P | P | P |
| C04AE01 | Ergoloid mesylates | x | x |  |  |  | M |  |  | M |  | M |
| C04AE02 | Nicergoline | x | x |  |  |  |  |  |  | M | M | M |
| C04AE04 | Dihydroergocristine | x | x |  |  |  |  |  |  | M |  | M |
| C04AE54 | Dihydroergocristine, combinations | x | x |  |  |  |  |  |  | M |  | M |
| C04AX01 | Cyclandelate | x | x |  |  |  |  |  |  | M |  |  |
| C04AX07 | Vincamine | x | x |  |  |  |  |  |  | M |  | M |
| C04AX10 | Moxisylyte | x | x |  |  |  |  |  |  | M |  | M |
| C04AX17 | Vinburnine | x | x |  |  |  |  |  |  | M |  | M |
| C04AX20 | Buflomedil | x | x |  |  |  |  |  |  | M |  |  |
| C04AX21 | Naftidrofuryl | x | x |  |  |  |  |  |  | M | M | M |
| C05CA05 | Hidrosmin | x | x |  |  |  |  |  |  | M |  |  |
| C05CA07 | Escin (aescin) | x | x |  |  |  |  |  |  | M |  |  |
| C05CA51 | Rutoside combinations | x | x |  |  |  |  |  |  | M |  | M |
| C05CA54 | Troxerutin, combinations | x | x |  |  |  |  |  |  | M |  | M |
| C07AA02 | Oxprenolol | x | x |  |  |  |  | I |  | M |  |  |
| C07AA03 | Pindolol |  |  | P |  |  |  | I |  | P |  |  |
| C07AA05 | Propranolol |  |  |  |  |  |  | I |  | P |  |  |
| C07AA07 | Sotalol |  |  | P |  |  |  | I |  | P | P |  |
| C07AA12 | Nadolol | x | x |  |  |  |  | I |  | M |  |  |
| C07AG01 | Labetalol | x |  |  |  |  |  | I |  | I |  |  |
| C08CA04 | Nicardipine | x | x |  |  |  |  |  |  | M |  | M |
| C08CA05 | Nifedipine |  |  |  |  |  | P |  |  | P | P | P |
| C08DA01 | Verapamil |  |  | P |  |  |  | I |  | P |  |  |
| C08DB01 | Diltiazem |  |  | P |  |  |  | I |  | P |  |  |
| C10AD02 | Nicotinic acid | x | x |  |  |  |  |  |  | M |  |  |
| G03AC05 | Megestrol | x | x |  |  |  | M |  |  |  |  |  |
| G03BA02 | Methyltestosterone | x | x |  |  |  | M |  |  |  |  |  |
| G03BA03 | Testosterone |  |  |  |  |  | P |  |  |  |  |  |
| G03CA03 | Estradiol |  |  | P |  |  |  | P |  | P |  |  |
| G03CA04 | Estriol |  |  | P |  |  |  | P |  | P |  |  |
| G03CX01 | Tibolone |  |  | P |  |  |  | P |  | P |  |  |
| G03FA01 | Norethisterone and estrogen |  |  | P |  |  |  | P |  | P |  |  |
| G03FA12 | Medroxyprogesterone and estrogen |  |  | P |  |  |  | P |  | P |  |  |
| G03FA14 | Dydrogesterone and estrogen |  |  | P |  |  |  | P |  | P |  |  |
| G04BD02 | Flavoxate | x | x |  |  |  | M |  |  | M |  |  |
| G04BD04 | Oxybutynin |  |  | P | P |  | P |  |  | P | P | P |
| G04BD07 | Tolterodine |  |  | P | P |  | P |  | P | P | P | P |
| G04BD08 | Solifenacin |  |  | P | P |  | P |  | P | P | P | P |
| G04BD09 | Trospium |  |  | P |  |  | P |  |  | P |  |  |
| G04BD10 | Darifenacin |  |  |  | P |  | P |  |  | P |  |  |
| G04BD11 | Fesoterodine |  |  | P | P |  | P |  | P | P |  |  |
| G04BX01 | Magnesium hydroxide |  |  | P |  |  |  |  |  |  |  |  |
| G04CA03 | Terazosin | x | x |  |  |  | M |  |  | M | M |  |
| G04CA53 | Tamsulosin and solifenacin |  |  |  |  |  |  |  |  | P |  |  |
| H01AC01 | Growth hormone (somatropin) |  |  |  |  |  | P |  |  |  |  |  |
| H01BA02 | Desmopressin |  |  | P |  |  | P |  |  |  |  |  |
| H03AA05 | Desiccated Thyroid  (thyroid gland preparations) | x | x |  |  |  | M |  |  |  |  |  |
| J01MA01 | Ofloxacin | x | x |  |  |  |  |  |  | M |  |  |
| J01XE01 | Nitrofurantoin |  |  | P |  |  | P |  |  | I | P | P |
| M01AA01 | Phenylbutazone | x | x |  |  | M |  | I | I | M | M | M |
| M01AB01 | Indometacin |  |  | P |  | P | P | I | P | P | P | P |
| M01AB02 | Sulindac | x | x |  |  | M | M | I |  |  |  |  |
| M01AB03 | Tolmetin | x | x |  |  | M | M | I |  |  |  |  |
| M01AB05 | Diclofenac |  |  |  |  | P | P | I | P | P |  |  |
| M01AB08 | Etodolac | x | x |  |  | M | M | I |  |  |  |  |
| M01AB11 | Acemetacin | x | x |  |  | M |  | I | M | M | M |  |
| M01AB15 | Ketorolac |  |  |  |  | P | P | I | P | P |  |  |
| M01AB16 | Aceclofenac | x | x |  |  | M |  | I | M | M |  |  |
| M01AB55 | Diclofenac, combinations |  |  |  |  | P | P | I | P | P |  |  |
| M01AC01 | Piroxicam | x | x |  |  | M | P | I | M | M | M |  |
| M01AC05 | Lornoxicam | x | x |  |  | M |  | I | M | M |  |  |
| M01AC06 | Meloxicam |  |  |  |  | P | P | I | P | P | P |  |
| M01AE01 | Ibuprofen |  |  |  |  | P | P | I | P | I |  |  |
| M01AE02 | Naproxen |  |  |  |  | P | P | I | P | I |  |  |
| M01AE03 | Ketoprofen |  |  |  |  | P | P | I | P | P | P |  |
| M01AE04 | Fenoprofen | x | x |  |  | M | M | I | M |  |  |  |
| M01AE09 | Flurbiprofen | x | x |  |  | M |  | I | M | M |  |  |
| M01AE12 | Oxaprozin | x | x |  |  | M | M | I | M |  |  |  |
| M01AE17 | Dexketoprofen |  |  |  |  | P |  | I | P | P |  |  |
| M01AE51 | Ibuprofen, combinations |  |  |  |  | P |  | I | P | I |  |  |
| M01AE52 | Naproxen and esomeprazole |  |  |  |  | P | P | I | P | I |  |  |
| M01AG01 | Mefenamic acid |  |  |  |  | P | P | I | P | P |  |  |
| M01AG04 | Meclofenamic acid | x | x |  |  | M | M | I | M |  |  |  |
| M01AH01 | Celecoxib |  |  |  |  | P |  | I | P | P |  |  |
| M01AH05 | Etoricoxib |  |  |  |  | P |  | I | P | P | P |  |
| M01AX01 | Nabumetone |  |  |  |  | P | P | I | P | P |  |  |
| M03BA02 | Carisoprodol | x | x |  |  |  | M |  |  | M |  |  |
| M03BA03 | Methocarbamol | x | x |  |  |  | M |  |  | M |  | M |
| M03BB03 | Chlorzoxazone | x | x |  |  |  | M |  |  |  |  |  |
| M03BC01 | Orphenadrine |  |  | P | P |  | P |  |  | P |  |  |
| M03BC51 | Orphenadrine, combinations |  |  | P | P |  | P |  |  | P |  |  |
| M03BX01 | Baclofen |  |  | P |  |  |  |  |  | P | P | P |
| M03BX02 | Tizanidine |  |  | P |  |  |  |  |  | P |  |  |
| M03BX07 | Tetrazepam | x | x |  |  |  |  |  |  | M | M | M |
| M03BX08 | Cyclobenzaprine | x | x |  |  |  | M |  |  | M |  |  |
| M04AC01 | Colchicine | x^1^ |  |  |  |  |  |  |  | P |  |  |
| M05BX03 | Strontium ranelate |  |  | P |  |  |  |  |  | I |  |  |
| M09AA | Quinine and derivatives | x | x |  |  |  |  |  |  | M |  |  |
| N02AA01 | Morphine |  |  |  |  |  |  | P | I |  |  |  |
| N02AA05 | Oxycodone |  |  |  |  |  |  | P | I |  |  |  |
| N02AA55 | Oxycodone and naloxone |  |  |  |  |  |  | P | I |  |  |  |
| N02AB02 | Pethidine | x | x |  |  |  | M | M |  | M | M |  |
| N02AB02 | Fentanyl |  |  |  |  |  |  | P |  |  |  |  |
| N02AD01 | Pentazocine | x | x |  |  |  |  | M |  | M |  |  |
| N02AE01 | Buprenorphine |  |  |  |  |  |  | P |  |  |  |  |
| N02AG | Opioids in combination with antispasmodics | x | x |  | M |  |  |  | I |  |  |  |
| N02AJ06 | Codeine and paracetamol |  |  | P | P | P |  |  |  |  |  |  |
| N02AJ08 | Codeine and ibuprofen |  |  | P | P |  |  |  |  |  |  |  |
| N02AJ13 | Tramadol and paracetamol |  |  | P | P |  |  | P |  | P |  |  |
| N02AJ14 | Tramadol and dexketoprofen |  |  | P | P |  |  | P |  | P |  |  |
| N02AX02 | Tramadol |  |  | P | P |  |  | P |  | P |  |  |
| N02BA01 | Acetylsalicylic acid |  |  | P |  |  | I | I |  | I |  |  |
| N02BA11 | Diflunisal | x | x |  |  |  | M |  |  |  |  |  |
| N02BA51 | Acetylsalicylic acid, combinations excluding psycholeptics |  |  | P |  |  |  | I |  | I |  |  |
| N02CA01 | Dihydroergotamine | x | x |  |  |  |  |  |  |  | M | M |
| N02CA02 | Ergotamine | x | x |  |  |  |  |  |  | M | M |  |
| N02CC | Triptanes (e.g. Sumatriptan, Eletriptan, Naratriptan, Zolmitriptan) | x |  |  |  |  |  |  |  | I |  |  |
| N03AA01 | Methylphenobarbital | x | x |  |  |  | M |  |  |  |  |  |
| N03AA02 | Phenobarbital | x | x |  |  |  | M |  |  | M | M |  |
| N03AB02 | Phenytoin |  |  | P |  |  |  |  |  | P |  |  |
| N03AE01 | Clonazepam |  |  | P |  |  | P |  |  | P |  |  |
| N03AF01 | Carbamazepine |  |  |  |  |  |  |  |  | P |  |  |
| N03AX11 | Topiramate |  |  |  |  |  |  |  |  | P |  |  |
| N04AA01 | Trihexyphenidyl | x | x |  | M |  | M |  |  | M |  | I |
| N04AA02 | Biperiden |  |  | P | P |  |  |  | P | P |  | I |
| N04AA12 | Tropatepine | x | x |  |  |  |  |  |  | M |  | I |
| N04AB02 | Orphenadrine | x | x |  |  |  |  |  | M |  |  |  |
| N04AC01 | Benzatropine | x | x |  |  |  | M |  |  | M |  |  |
| N04BB01 | Amantadine | x^1^ |  |  |  |  |  |  |  | P |  |  |
| N04BC01 | Bromocriptine |  |  |  |  |  |  |  |  | P |  |  |
| N04BC02 | Pergolide | x | x |  |  |  |  |  |  | M |  |  |
| N04BC03 | Dihydroergocryptine | x | x |  |  |  |  |  |  | M | M | M |
| N04BC04 | Ropinirole |  |  |  |  |  |  |  |  | P |  |  |
| N04BC05 | Pramipexole |  |  |  |  |  |  |  |  | P |  |  |
| N04BC06 | Cabergoline |  |  |  |  |  |  |  |  | P |  |  |
| N04BC08 | Piribedil | x | x |  |  |  |  |  |  | M |  | M |
| N04BC09 | Rotigotine | x |  |  |  |  |  |  |  | I |  |  |
| N04BD01 | Selegiline | x |  |  |  |  |  |  |  | I |  |  |
| N05AA01 | Chlorpromazine |  |  |  |  |  | P | P |  | P |  | P |
| N05AA02 | Levomepromazine |  |  | P | P |  | P | P | P | P | P | P |
| N05AA04 | Acepromazine | x | x |  |  |  | M | M |  | M |  | M |
| N05AA06 | Cyamemazine | x | x |  |  |  | M | M |  | M |  | M |
| N05AB02 | Fluphenazine | x | x |  |  |  | M | M |  | M | M | M |
| N05AB03 | Perphenazine |  |  | P |  |  | P | P |  | P | P | P |
| N05AB04 | Prochlorperazine |  |  | P | P |  | P | P |  | P |  |  |
| N05AB06 | Trifluoperazine | x | x |  |  |  | M | M |  | M |  |  |
| N05AC01 | Periciazine |  |  |  |  |  | P | P |  | P |  | P |
| N05AC02 | Thioridazine | x | x |  |  |  | M | M |  | M | M |  |
| N05AC04 | Pipotiazine | x | x |  |  |  | M | M |  | M |  | M |
| N05AD01 | Haloperidol |  |  | P |  |  | P | P | P | I | I |  |
| N05AD08 | Droperidol |  |  |  |  |  | P | P |  | P |  |  |
| N05AE03 | Sertindole |  |  |  |  |  | P | P |  | P |  |  |
| N05AE04 | Ziprasidone |  |  |  |  |  | P | P |  | P |  |  |
| N05AF01 | Flupentixol |  |  |  |  |  | P | P | P | P |  |  |
| N05AF03 | Chlorprothixene |  |  | P | P |  | P | P | P | P |  |  |
| N05AF05 | Zuclopenthixol |  |  |  |  |  | P | P | P | P |  |  |
| N05AG02 | Pimozide | x | x |  |  |  | M | M |  | M |  |  |
| N05AH01 | Loxapine | x | x |  |  |  | M | M |  |  |  |  |
| N05AH02 | Clozapine |  |  | P | P |  | P | P |  | P | P |  |
| N05AH03 | Olanzapine |  |  |  |  |  | P | P | P | I | I |  |
| N05AH04 | Quetiapine |  |  |  |  |  | P | P | P |  |  |  |
| N05AN01 | Lithium |  |  | P |  |  | P | P |  | P |  |  |
| N05AX08 | Risperidone |  |  |  |  |  | P | P | I | I |  |  |
| N05AX12 | Aripiprazole |  |  |  |  |  | P | P | P | P |  |  |
| N05BA01 | Diazepam |  |  | P | P | P | P | P | P | P | P | P |
| N05BA02 | Chlordiazepoxide |  |  |  |  |  | P | P |  | P | P | P |
| N05BA03 | Medazepam | x | x |  |  |  |  | M |  | M | M | I |
| N05BA04 | Oxazepam |  |  |  |  | I | P | P | I | I | I | I |
| N05BA05 | Potassium clorazepate | x | x |  |  |  | M | M |  | M | M | M |
| N05BA06 | Lorazepam |  |  |  |  |  | P | P |  | I | I | I |
| N05BA08 | Bromazepam | x | x |  |  |  |  | M |  | M | M | M |
| N05BA09 | Clobazam |  |  |  |  |  |  | P |  | P | P | P |
| N05BA11 | Prazepam | x | x |  |  |  |  | M |  | M | M | M |
| N05BA12 | Alprazolam |  |  | P |  |  | P | P |  | P | P | I |
| N05BA13 | Halazepam | x | x |  |  |  |  | M |  | M |  | I |
| N05BA16 | Nordazepam | x | x |  |  |  |  | M |  | M |  | M |
| N05BA18 | Ethyl loflazepate | x | x |  |  |  |  | M |  | M |  | M |
| N05BA21 | Clotiazepam | x | x |  |  |  |  | M |  | I |  | I |
| N05BB01 | Hydroxyzine |  |  | P | P | P | P | P |  | P | P | P |
| N05BC01 | Meprobamate | x | x |  |  |  | M |  |  | M |  | I |
| N05CA01 | Pentobarbital | x | x |  |  |  | M |  |  |  |  |  |
| N05CA02 | Amobarbital | x | x |  |  |  | M |  |  |  |  |  |
| N05CA03 | Butobarbital | x | x |  |  |  | M |  |  |  |  |  |
| N05CA06 | Secobarbital | x | x |  |  |  | M |  |  |  |  |  |
| N05CC01 | Chloralhydrate | x | x |  |  | M |  |  |  | M | M |  |
| N05CD01 | Flurazepam | x | x |  |  | M | M | M |  | M | M | I |
| N05CD02 | Nitrazepam |  |  | P | P | P |  | P | P | P | P | P |
| N05CD03 | Flunitrazepam | x | x |  | M | M |  | M |  | M | M | M |
| N05CD04 | Estazolam | x | x |  |  | M | M | M |  | M |  | M |
| N05CD05 | Triazolam |  |  | P |  | P | P | P |  | P | P | I |
| N05CD06 | Lormetazepam | x | x |  |  | M |  | M |  | I | I | I |
| N05CD07 | Temazepam |  |  |  |  | P | P | P |  | P | P | I |
| N05CD08 | Midazolam |  |  | P |  | P |  | P |  | P |  | I |
| N05CD09 | Brotizolam | x | x |  |  | M |  | M |  | I | I | I |
| N05CD10 | Quazepam | x | x |  |  | M | M | M |  | M |  | I |
| N05CD11 | Loprazolam | x | x |  |  | M |  | M |  | I |  | I |
| N05CF01 | Zopiclone |  |  |  |  | P |  | P | I | I | I | I |
| N05CF02 | Zolpidem |  |  | P |  | P | P | P | I | I | I | I |
| N05CF03 | Zaleplon | x | x |  |  | M | M | M |  | I | I |  |
| N05CF04 | Eszopiclone | x | x |  |  | M | M |  |  |  |  |  |
| N05CM02 | Chlomethiazole | x | x |  |  | M |  |  |  | M |  |  |
| N05CM06 | Propiomazine | x | x |  | M | M |  |  |  | M |  |  |
| N06AA01 | Desipramine | x | x |  |  |  | M | M |  | M |  |  |
| N06AA02 | Imipramine | x | x |  |  |  | M | M | M | M | M | M |
| N06AA04 | Clomipramine |  |  | P | P | P | P | P | P | P | P | P |
| N06AA06 | Trimipramine |  |  | P |  | P | P | P |  | P | P | P |
| N06AA09 | Amitriptyline |  |  | P | P | P | P | P | P | P | P | P |
| N06AA10 | Nortriptyline |  |  |  | P | P | P | P | P | P |  |  |
| N06AA11 | Protriptyline | x | x |  |  |  | M | M |  |  |  |  |
| N06AA12 | Doxepin |  |  | P |  | P | I | P |  | P | P | P |
| N06AA16 | Dosulepin | x | x |  |  |  |  | M |  | M |  | M |
| N06AA17 | Amoxapine | x | x |  |  |  | M | M |  | M |  | M |
| N06AA21 | Maprotiline | x | x |  | M |  |  | M |  | M | M | M |
| N06AB03 | Fluoxetine |  |  | P |  |  |  |  |  | P | P |  |
| N06AB05 | Paroxetine |  |  | P |  |  | P |  |  | P |  |  |
| N06AB08 | Fluvoxamine |  |  | P |  |  |  |  |  | P |  |  |
| N06AF04 | Tranylcypromine | x | x |  |  |  |  |  |  | M | M |  |
| N06AG02 | Moclobemide |  |  | P |  |  |  |  |  |  |  |  |
| N06AX05 | Trazodone |  |  | P |  |  |  |  |  |  |  |  |
| N06AX12 | Bupropion | x^1^ |  |  |  |  |  |  |  | P |  |  |
| N06AX16 | Venlafaxine |  |  |  |  |  |  |  |  | P |  |  |
| N06AX18 | Reboxetine | x^1^ |  |  |  |  |  |  |  | P |  |  |
| N06BA04 | Methylphenidate | x^1^ |  |  |  |  |  |  |  | P |  |  |
| N06BX03 | Piracetam |  |  |  |  |  |  |  |  | P | P | P |
| N06CA01 | Amitriptyline and psycholeptics |  |  | P |  |  |  |  |  | P |  |  |
| N06DX02 | Ginkgo biloba/folium | x | x |  |  |  |  |  |  | M |  | M |
| N07AB02 | Bethanechol | x | x |  |  |  |  |  |  | M |  |  |
| N07BC01 | Buprenorphine |  |  |  |  |  |  | P |  |  |  |  |
| N07BC02 | Methadone |  |  |  |  |  |  | P |  | P |  |  |
| N07BC06 | Diamorphine | x | x |  |  |  |  | M |  |  |  |  |
| N07BC51 | Buprenorphine, combinations |  |  |  |  |  |  | P |  |  |  |  |
| no ATC | Guanabenz | x | x |  |  |  | M |  |  |  |  |  |
| no ATC | Butalbital | x | x |  |  |  | M |  |  |  |  |  |
| no ATC | Aceprometazine | x | x |  |  |  |  |  |  | M |  | M |
| no ATC | Metaxalone | x | x |  |  |  | M |  |  |  |  |  |
| P01BC01 | Quinine | x | x |  |  |  |  |  | M |  |  |  |
| R01BA01 | Phenylpropanolamine |  |  |  |  |  |  |  |  | P |  |  |
| R01BA52 | Pseudoephedrine |  |  |  |  |  |  |  |  | P |  |  |
| R03CC03 | Terbutaline | x | x |  |  |  |  |  |  | M |  |  |
| R03DA04 | Theophylline |  |  | P |  |  |  | P |  | P |  |  |
| R03DA54 | Theophylline, combinations |  |  | P |  |  |  | P |  | P |  |  |
| R05DA01 | Ethylmorphine |  |  | P |  |  |  |  |  | P |  |  |
| R05DA04 | Codeine | x | x |  | M |  |  |  |  | I |  |  |
| R05DA09 | Dextromethorphan |  |  | P |  |  |  |  |  | P |  |  |
| R05FA02 | Opium derivatives and expectorants |  |  | P |  |  |  |  |  |  |  |  |
| R06AA02 | Diphenhydramine | x | x |  | M |  | M | M |  | M | M | M |
| R06AA04 | Clemastine | x | x |  | M |  | M | M |  | M | M |  |
| R06AA08 | Carbinoxamine | x | x |  |  |  | M | M |  | M |  | M |
| R06AA09 | Doxylamine |  |  | P |  |  | P | P |  | P | P | P |
| R06AB01 | Brompheniramine | x | x |  |  |  | M | M |  | M |  | M |
| R06AB02 | Dexchlorpheniramine | x | x |  | M | M | M | M |  | M |  | M |
| R06AB03 | Dimetindene | x | x |  |  |  |  |  |  | M | M |  |
| R06AB04 | Chlorphenamine | x | x |  | M |  | M | M |  | M | M | M |
| R06AB05 | Pheniramine | x | x |  |  |  |  |  |  | M |  | M |
| R06AB06 | Dexbrompheniramine | x | x |  |  |  | M | M |  |  |  |  |
| R06AB52 | Dexchlorpheniramine, combinations | x | x |  |  |  |  |  |  | M |  | M |
| R06AC01 | Mepyramine | x | x |  |  |  | M | M |  |  |  |  |
| R06AC04 | Tripelennamine | x | x |  |  |  |  | M |  | M |  |  |
| R06AD01 | Alimemazine | x | x |  | M | M |  | M |  | M |  | M |
| R06AD02 | Promethazine | x | x |  | M | M | M | M | I | M |  | M |
| R06AD03 | Thiethylperazine | x | x |  | M |  |  | M |  |  |  |  |
| R06AD07 | Mequitazine | x | x |  |  |  |  | M |  | M |  | M |
| R06AD08 | Oxomemazine | x | x |  |  |  |  | M |  | M |  | M |
| R06AE01 | Buclizine | x | x |  |  |  |  | M |  | M |  | M |
| R06AE03 | Cyclizine |  |  | P |  |  |  | P |  | P |  |  |
| R06AE05 | Meclozine |  |  | P | P |  | P | P |  | P |  | P |
| R06AX02 | Cyproheptadine | x | x |  | M |  | M | M |  | M |  | M |
| R06AX07 | Triprolidine | x | x |  |  |  | M | M |  | M | M | M |
| R06AX12 | Terfenadine | x | x |  |  |  |  |  |  | M |  |  |
| R06AX22 | Ebastine | x |  |  |  |  |  |  |  | I |  |  |
| R06AX23 | Pimethixene | x | x |  |  |  |  |  |  | M |  | M |

1: misinterpreted from the EU(7)-PIM list and not included in the data collection; I: excluded statement in order to form a table of medications that should be avoided by older people in most circumstances: statements considering concurrent use of two or more drugs, specific medical condition, restriction of treatment duration or dose, limited research evidence or experience among older people; M: potentially inappropriate medication not available on Finnish market; NH: nursing home; NORGEP: Norwegian General Practice; P: considered as potentially inappropriate medication; PIM: potentially inappropriate medication; STOPP/START: Screening Tool of Older Persons’ Potentially Inappropriate Prescriptions and Screening Tool to Alert to Right Treatment
