# Supplementary material for: Prevalence of potentially inappropriate medication use in older population: comparison of the Finnish Meds75+ database with eight published criteria
Source: BMC Geriatr. 2023 Mar 10;23:139. doi: 10.1186/s12877-022-03706-z (PMC9999502; doi:10.1186/s12877-022-03706-z)
Supplement: Supplementary file 2 — Additional file 2. Proportion of users of the most common medicine classes/substances among people using PIMs (total = 497,663). [file 12877_2022_3706_MOESM2_ESM.docx]

Prevalence of potentially inappropriate medication use in older population: comparison of the Finnish Meds75+ database with eight published criteria

**Journal name:** BMC Geriatrics

**Author names:** Paulamäki Jasmin, Jyrkkä Johanna, Hyttinen Virva, Jämsen Esa

**Corresponding author:** Jasmin Paulamäki, Faculty of Medicine and Health Technology, Clinical Medicine, Tampere University, Tampere, Finland. Email: jasmin.paulamaki@tuni.fi

**Description of the data:** Proportion of users of the most commonly used medicine classes and individual drug substances considered as PIMs among people with at least one PIM in use during the observation period (total number of persons = 497,663).

**Abbreviations:**  ^1^: the top three individual drug substances of each medicine classes per each nine criteria are presented; ^2^: potentially inappropriate medications according to the summary of nine criteria. Even if the same medicine class is included in several PIM criteria, the substances considered as PIMs may differ between the criteria, leading to variation in the reported percentages; *NH*: nursing home; *NORGEP*: Norwegian General Practice; *PIM*: potentially inappropriate medication; *STOPP/START*: Screening Tool of Older Persons’ Potentially Inappropriate Prescriptions and Screening Tool to Alert to Right Treatment

**Appendix 2.** Proportion of users of the most common medicine classes/substances among people using PIMs (total=497,663).

| **ATC code and medicine subgroup** | **All criteria considered^2^ (%)** | **Meds75+ database [13] (%)** | **Beers criteria [24] (%)** | **EU(7)-PIM list [23] (%)** | **Indicators for Quality of Drug Therapy in the elderly [12] (%)** | **Laroche criteria [8] (%)** | **NORGEP-NH [25] (%)** | **PRISCUS list [22] (%)** | **Red-Yellow-Green list [26] (%)** | **STOPP(/START) [27] (%)** |
| --- | --- | --- | --- | --- | --- | --- | --- | --- | --- | --- |
| A02B Drugs for peptic ulcer and gastro-oesophageal reflux disease | 46.8 | 0.8 | 46.4 |  |  |  |  |  |  |  |
|  |  | Alginic acid (0.7) Sucralfate (0.1) | Pantoprazole (33.0) Esomeprazole (8.6) Omeprazole (4.3) |  |  |  |  |  |  |  |
| N02A Opioids | 38.1 | 20.5 |  | 6.6 | 20.5 |  | 16.0 |  |  | 28.4 |
|  |  | Codeine (16.0)  Tramadol (6.6) |  | Tramadol (6.6) | Codeine (16.0) Tramadol (6.6) |  | Combination of codeine and paracetamol (16.0) |  |  | Oxycodone (15.6) Buprenorphine (12.4)  Tramadol (6.6) |
| C03C High-ceiling diuretics/Loop diuretics | 33.5 |  |  |  |  |  |  |  |  | 33.5 |
|  |  |  |  |  |  |  |  |  |  | Furosemide (33.5) |
| M01A Anti-inflammatory products, non-steroids | 30.1 | 0.4 | 22.8 | 16.0 |  | 0.4 | 30.1 | 12.1 | 30.1 |  |
|  |  | Indomethacin (0.4) | Ibuprofen (15.3) Diclofenac (4.3) Naproxen (3.1) | Etoricoxib (9.7) Diclofenac (4.3) Meloxicam (2.1) |  | Indomethacin (0.4) | Ibuprofen (15.3) Etoricoxib (9.7) Diclofenac (4.3) | Etoricoxib (9.7) Meloxicam (2.1) Ketoprofen (0.5) | Ibuprofen (15.3) Etoricoxib (9.7) Diclofenac (4.3) |  |
| N05C Hypnotics and sedatives | 23.4 | 3.8 | 9.7 | 7.0 | 0.3 | 0.3 | 23.4 | 6.9 | 0.3 | 23.4 |
|  |  | Zolpidem (3.3)  Nitrazepam (0.3) Midazolam (0.2) | Temazepam (6.6) Zolpidem (3.3) Triazolam (0.1) | Temazepam (6.6) Nitrazepam (0.3) Midazolam (0.2) | Nitrazepam (0.3) | Nitrazepam (0.3) | Zopiclone (14.7) Temazepam (6.6)  Zolpidem (3.3) | Temazepam (6.6) Nitrazepam (0.3) Triazolam (0.1) | Nitrazepam (0.3) | Zopiclone (14.7) Temazepam (6.6) Zolpidem (3.3) |
| N05B Anxiolytics | 17.6 | 6.2 | 17.5 | 6.3 | 5.4 | 5.5 | 5.4 | 6.3 | 3.0 | 17.6 |
|  |  | Diazepam (3.0) Hydroxyzine (2.5) Alprazolam (0.8) | Oxazepam (10.2) Diazepam (3.0) Lorazepam (3.0) | Diazepam (3.0) Hydroxyzine (2.5) Alprazolam (0.8) | Diazepam (3.0) Hydroxyzine (2.5) | Diazepam (3.0) Hydroxyzine (2.5) Chlordiazepoxide (0.1) | Diazepam (3.0) Hydroxyzine (2.5) | Diazepam (3.0) Hydroxyzine (2.5) Alprazolam (0.8) | Diazepam (3.0) | Oxazepam (10.2) Diazepam (3.0) Lorazepam (3.0) |
| N05A Antipsychotics | 14.4 | 1.9 | 14.4 | 1.4 | 0.8 | 0.6 |  | 0.7 | 9.0 | 14.4 |
|  |  | Haloperidol (0.8) Levomepromazine (0.3)  Prochlorperazine (0.3) | Quetiapine (7.0) Risperidone (6.8) Olanzapine (1.5) | Levomepromazine (0.3)  Prochlorperazine (0.3)  Perphenazine (0.3) | Levomepromazine (0.3) Prochlorperazine (0.3)  Chlorprothixene (0.1) | Levomepromazine (0.3) Perphenazine (0.3) Propericiazine (0.03) |  | Levomepromazine (0.3)  Perphenazine (0.3) Clozapine (0.1) | Quetiapine (7.0) Olanzapine (1.5) Haloperidol (0.8) | Quetiapine (7.0) Risperidone (6.8) Olanzapine (1.5) |

^1^: the top three individual drug substances of each medicine classes per each nine criteria are presented; ^2^: potentially inappropriate medications according to the summary of nine criteria. Even if the same medicine class is included in several PIM criteria, the substances considered as PIMs may differ between the criteria, leading to variation in the reported percentages; *NH*: nursing home; *NORGEP*: Norwegian General Practice; *PIM*: potentially inappropriate medication; *STOPP/START*: Screening Tool of Older Persons’ Potentially Inappropriate Prescriptions and Screening Tool to Alert to Right Treatment
